# Analysis of results from the Joint External Evaluation: examining its strength and assessing for trends among participating countries

**DOI:** 10.7189/jogh.08.020416

**Published:** 2018-12

**Authors:** Vin Gupta, John D Kraemer, Rebecca Katz, Ashish K Jha, Vanessa B Kerry, Jussi Sane, Jukka Ollgren, Mika O Salminen

**Affiliations:** 1 Division of Pulmonary and Critical Care Medicine, Brigham & Women’s Hospital, Boston, Massachusetts, USA; 2 Harvard Global Health Institute, Harvard University, Cambridge, Massachusetts, USA; 3 Institute for Health Metrics and Evaluation, Seattle, Washington, USA; 4 Georgetown University, Department of Health Systems Administration, Washington, D.C., USA; 5 Georgetown University, Center for Global Health Science and Security, Washington, D.C., USA; 6 Department of Health Policy, Harvard T.H. Chan School of Public Health, Boston, Massachusetts, USA; 7 Department of Global Health and Social Medicine, Harvard Medical School, Boston, Massachusetts, USA; 8 MGH Global Health, Massachusetts General Hospital, Boston, Massachusetts, USA; 9 Seed Global Health, Boston, Massachusetts, USA; 10 Department for Health Security, National Institute for Health and Welfare (THL), Helsinki, Finland

## Abstract

**Background:**

The Joint External Evaluation (JEE) is part of the World Health Organization’s (WHO) new process to help countries assess their ability to prevent, detect and respond to public health threats such as infectious disease outbreaks, as specified by the International Health Regulations (IHR). How countries are faring on these evaluations is not well known and neither is there any previous assessment of the performance characteristics of the JEE process itself.

**Methods:**

We obtained JEE data for 48 indicators collectively across 19 technical areas of preparedness for 55 countries. The indicators are scored on a 1 to 5 scale with 4 indicating demonstrated capacity. We created a standardized JEE index score representing cumulative performance across indicators using principal components analysis. We examined the state of performance across all indicators and then examined the relationship between this index score and select demographic and health variables to better understand potential drivers of performance.

**Results:**

Among our study cohort, the median performance on 43 of the 48 (89.6%) indicators was less than 4, suggesting that countries were failing to meet demonstrated capacity on these measures. The two weakest indicators were related to antimicrobial resistance (median score = 1.0, interquartile range = 1.0-2.0) and biosecurity response (median score = 2.0, interquartile range = 2.0-3.0). JEE index scores correlated with various metrics of health outcomes (life expectancy, under-five year mortality rate, disability-adjusted life years lost to communicable diseases) and with standard measures of social and economic development that enable public health system performance in the total sample, but in stratified analyses, these relationships were much weaker in the AFRO region.

**Conclusions:**

We find large variations in JEE scores among countries and WHO regions with many nations still unprepared for the next disease outbreak with pandemic potential The strong correlations between JEE performance and metrics of both health outcomes and health systems’ performance suggests that the JEE is likely accurately measuring the strength of IHR-specific, public health capabilities.

Building global capacity to prevent, detect and respond to epidemics with pandemic potential has proven difficult [1,2]. The revised International Health Regulations (IHR), adopted in 2005, obligated all Member States of the World Health Assembly to build capacities to detect, assess, notify, and respond to potential public health emergencies in a timely manner [3,4]. Weak accountability mechanisms to ensure compliance and lack of dedicated resources have complicated these efforts and are widely seen as part of the explanation for the poor local and global response to the Ebola outbreak in western Africa [5].

Preparing for population level health threats, such as infectious disease outbreaks, requires sustained investments in certain public health functions, including preventive interventions (eg, immunization programs, food and waterborne disease control measures), physical infrastructure (eg, laboratory and surveillance capabilities), trained personnel (eg, health professionals, epidemiologists) [6-8], and ongoing commitments to resources for supplies, maintenance, and training [9]. Given competing resources, relatively few countries have made consistent investments in these areas and a majority have been unable to meet their obligations under the IHR. Major donors of health aid have often focused their attention elsewhere as well [10,11]. While investment in these population level preventive functions is generally regarded as highly cost-effective, investments in curative health services are frequently prioritized due to high demand and greater visibility [12].

In 2010, the World Health Organization (WHO) identified 13 core capacities derived from Annex 1 of the IHR for the purpose of monitoring capacity building efforts and compliance. These core capacities were used by countries to complete self-assessments and self-reporting to WHO, but these assessments were criticized for lack of transparency and not representing the true measure of capacity for health security within countries [13,14]. Although variation exists, analyses of the reported self-assessed country data suggests probable overestimation of real capacity in many areas of the world (Tsai and Katz, submitted manuscript). Following the 2014-2016 Ebola outbreak in West Africa and the spread of Middle Eastern Respiratory Syndrome (MERS) to the Republic of Korea in 2015, and in response to recommendations provided by the IHR review committee [13], the WHO developed (through a regional consultation process) a new approach to monitoring and evaluation of the IHR [15-17]. As part of this new approach, the WHO adopted the Joint External Evaluation (JEE) process and tool [18] in January 2016 for broad voluntary use in assessing the ability of a nation to prevent, detect and respond to a disease of pandemic potential [19-21].

The JEE tool includes 19 technical areas with 48 indicators. A globally standardized process, the first step of a JEE is a self-assessment by an internal country team; these results are then compared and validated against a separate external evaluation conducted by a team of independent substance experts. Ultimately, the JEE results are supposed to contribute to the development of costed and financed multisectoral national action plans designed to address any prioritized gaps in a country’s capacity to prevent, detect and respond to public health threats [22]. Now two years into its implementation, there has been scant examination of country-level results from the JEE.

In this study, we used the most recent data available on JEE assessments to answer two questions: First, how are assessed countries performing across all indicators and specific technical areas within the JEE and what are the regional variations in performance? Second, as a new process, do JEE scores correlate in expected ways with population-level health outcomes (results of public health capacities) and inputs (determinants of public health capacities). In other words, does the JEE accurately measure the core public health capacities it intends to assess?

## METHODS

### Data sources

Joint External Evaluation (JEE) data was extracted from April 1, 2017 to March 4, 2018; 55 countries’ data was available (Appendix S1 in **Online Supplementary Document(Online Supplementary Document)**). Data was gathered from the WHO Strategic Partnership Portal (https://extranet.who.int/spp/ihr-monitoring-evaluation) [23], which provides a full overview and continuous updates of JEEs, published mission reports and other information on WHO supported activities to strengthen IHR implementation. The process for assessment via the JEE has been fully described previously [24].

Online databases from the Institute for Health Metrics and Evaluation (IHME) and the World Bank provided relevant health, demographic, and economic descriptive statistics for each country in our study sample. Global burden of disease (GBD) data from 2015, as measured in disability-adjusted life years (DALYs) per 100 000 persons, was available from the IHME (http://www.healthdata.org/results/data-visualizations). The most recent figures on population size, gross domestic product (GDP) per capita, public health expenditures as a percentage of GDP, life expectancy, under 5-year mortality, and density of skilled health professionals were available from the World Bank (http://databank.worldbank.org/data/).

### Definitions

The JEE tool is divided into 19 technical areas (representing public health functions relevant to health security) represented by 48 separate indicators (1 to 5 per technical area) (Appendix S2 in **Online Supplementary Document(Online Supplementary Document)**). Scores for each of the 19 technical areas represent the mean of the scores for the indicators of that area. Each indicator in the JEE is scored on a five-point ordinal scale. A score of 1 reflects no pertinent capacity, 2 reflects limited capacity, 3 notes developed capacity, 4 connotes demonstrated capacity, and a score of 5 reflects sustainable capacity (Appendix S3 in **Online Supplementary Document(Online Supplementary Document)**). For the purposes of our analysis, we identified a score of 4 as representing a minimum level of desired target performance, as we believe this indicates functionality and, at least, medium-term sustainability in a specific indicator.

To assess concordance with JEE performance, we chose three indicators that measure national health outcomes and three which measure inputs that enable public health system performance. The measures chosen to examine correlations with selected health outcomes were life expectancy at birth (years), under 5-year mortality rate (per 1000 live births) and disability-adjusted life years lost (DALYs) due to communicable disease (per 100 000 persons). These were selected as general population health, childhood health and communicable disease burden indicators. The three indicators that represent enabling factors were GDP per capita (in US$), public health expenditures as a percentage of GDP, and density of skilled health professionals (per 10 000 persons). Other indicators were available, but for the purposes of conciseness we decided to limit our study to one indicator each representing various aspects of adult, childhood and communicable disease-specific population health statuses, general economic development levels, investment in public health, and the availability of health professionals. Indicators were subjectively chosen based on frequent use for various international benchmarking analyses [25,26].

### Analyses

We produced basic descriptive statistics (medians and interquartile ranges) for various baseline demographic variables both for the overall data set and stratified by WHO region, merging the regional offices for Europe (EURO) and the Americas (PAHO) because few JEEs have been conducted in those regions. We assessed differences in these variables across regions using the Kruskall-Wallis test. Similar descriptive statistics were reported for each of the 48 indicators comprising the 19 technical areas of the JEE. The distribution of medians for these 48 indicators are presented in histogram format, with results stratified by WHO region.

In order to assess how representative our 55-country sample was of all 196 WHO states parties, we assessed for differences among the above baseline variables between those countries that did and did not complete the JEE. These variables were: population, GDP per capita, life expectancy, public health expenditures as a share of GDP (%), under-5-year mortality rate, DALYs due to communicable diseases, and density of skilled health professionals. Differences in the distributions of the variables were examined using the Mann-Whitney U-test and results were stratified by WHO region.

Next, we examined whether JEE performance correlated with population health outcomes and enabling factors as expected. To do so, we first created a JEE index score by applying principal components analysis to a polychoric correlation matrix, which correctly handles ordinal variables, of the 19 individual technical area scores [27]. Using a scree plot, we determined that there was only one principal component (which explained 71% of the variance and which was the only component with an eigenvalue greater than 1). This component was retained; its score was calculated and then standardized for ease of interpretation. We then assessed correlations between the JEE index score and variables representing each enabling factor and health outcome using Spearman’s rank-order correlation and calculated bias-corrected and accelerated percentile confidence intervals using 10 000 bootstrap replicates [28]. We analyzed region-specific correlations for AFRO and EMRO, the regions with the largest samples, to assess whether there were contextual differences in associations.

We conducted descriptive analyses using Microsoft Excel; all other analyses used Stata version 15.1 (Stata Corp, College Station, TX, USA). Statistical code to replicate the analysis is included as Appendix S1 in **Online Supplementary Document(Online Supplementary Document)**.

### Patient involvement

This study did not involve patients in its design, nor were the development of outcome measures informed by patients’ experiences or preferences. Our institutions did not require human subjects review because the study involved no interaction with human subjects or use of their data.

## RESULTS

Fifty-five of the 196 IHR states parties have completed JEE assessments that were publically available for review as of March 4, 2018. Twenty-three (41.8%) of the analyzed countries are from the WHO Africa region, 13 from the Eastern Mediterranean (23.6%), eight from Europe (14.5%), five from Southeast Asia (9.1%), five (9.1%) from the Western Pacific, and one from the Americas (1.8%). Together, the 55-nation sample represents 25.8% (n = 1.88 billion) of the world population, and 69.1% (n = 38) are classified as low or lower-middle income by the World Bank (by comparison, 42.8% or n = 83 of all WHO-member countries are classified as lower or lower-middle income). The distribution of demographic, economic and health variables among these regions are reported in **Table 1**. Among our study sample, significant differences were observed across regions, with countries in AFRO reporting the poorest levels of performance across these various factors. Within each region, the countries that have undergone the JEE were observed, with two exceptions, to not have significant differences across these baseline variables when compared to those countries who have yet to undergo the assessment (**Table 2**).

**Table 1:** Differences in demographic and health variables* across all countries globally who have undergone the JEE, stratified by WHO region

	AFRO	EMRO	PAHO+EURO	SEARO	WPRO	*P*
**Variable: Median (IQR)**	**Variable: Median (IQR)**	**Variable: Median (IQR)**	**Variable: Median (IQR)**	**Variable: Median (IQR)**
Number of countries	23	13	9	5	5	
Population*	16.1 (5.2-40.1)	11.3 (5.9-33.7)	5.5 (2.9-6.0)	52.4 (21.0-68.7)	11.1 (4.8-53.6)	0.23
GDP per capita†	890 (645-1350)	4100 (2510-20 730)	13700 (3950-40 350)	3840(1210-5810)	2130 (1635-3050)	<0.001
Public health expenditures (% GDP)	2.4 (1.8-3.2)	3.0 (1.9-3.3)	3.7 (2.9-7.3)	2.0 (1.0-5.6)	1.9 (1.1-3.2)	0.29
Life expectancy (years)	60.8 (55.4-64.1)	74.5 (66.4-77.3)	78.0 (74.1-81.1)	74.6 (72.0-75.0)	69.2 (67.6-72.8)	<0.001
Under 5-y mortality rate‡	69.9 (49.4-93.7)	14.5 (8.3-70.1)	7.9 (4.1-14.1)	12.3 (9.8-37.6)	25.6 (22.1-47.7)	<0.001
DALYs due to communicable disease§	34 730 (28 220-48 540)	4200 (2860-19 350)	2660 (1310-3720)	5800 (4130-12 770)	10 570(6180-17 140)	<0.001
Density of skilled health professionals¶	6.9 (4.6-10.4)	46.2 (14.9-49.4)	98.2 (77.5-122.8)	24.7 (15.0-24.8)	17.7 (10.8-44.8)	<0.001

JEE – Joint External Evaluation, AFRO – Africa Regional Office, PAHO – Pan-American Health Organization, EMRO – Eastern Mediterranean Regional Office, SEARO – Southeast Asia Regional Office, WPRO – Western Pacific Regional Office, STDEV – standard deviation, GDP – gross domestic product, DALY – disability adjusted life years

*Millions of persons.

†United States dollars (US$).

‡Per 1000 live births.

§Per 100 000 persons.

¶Per 10 000 persons.

**Table 2:** Differences in select demographic and health variables between countries who did and did not complete the JEE assessment, stratified by WHO regional grouping

WHO region	No. of countries	Variable median (IQR)													
Population*	***P***	**GDP per capita†**	***P***	**Public health expenditures (% GDP)**	***P***	**Life expectancy (years)‡**	***P***	**Under 5-y mortality rate**	***P***	**DALYs due to CD**§	***P***	**Density of skilled professionals**	***P***	
**AFRO:**																
JEE	23	11.9 (2.2-24.2)	0.02	830.0 (587.5-1313.9)	0.28	2.3 (1.7-3.2)	0.11	60.5 (55.4-64.1)	0.48	69.9 (49.6-93.7)	0.25	34.7 (28.2-48.5)	0.65	6.1 (4.1-10.1)	0.13	
Non-JEE	24	6.2 (1.3-17.8)		1320.0 (574.0-4951.0)		2.9 (2.2-3.8)		61.0 (57.3-64.7)		67.4 (42.7-92.6)		39.0 (32.1-45.7)		10.5 (6.8-15.9)		
**EMRO:**																
JEE	13	11.3 (5.9-33.7)	0.92	4096.0 (2514.0-20730.0)	0.66	3.0 (2.0- 3.4)	0.91	74.5 (66.4-77.3)	0.22	14.5 (8.3-70.1)	0.71	4.2 (2.9-19.4)	0.56	46.2 (14.9-49.4)	0.61	
Non-JEE	9	18.7 (4.4-36.1)		3547.0 (1862.0-4974.0)		2.7 (1.9-3.5)		71.3 (69.6-73.1)		21.1 (13.4-32.0)		4.9 (3.6-15.1)		28.5 (16.6-52.6)		
**PAHO+EURO:**																
JEE	9	5.5 (2.9- 6.0)	0.71	13670.0 (3950.0-40360.0)	0.98	3.7 (2.9-7.3)	0.75	78.0 (74.1-81.1)	0.81	7.9 (4.1-14.1)	0.89	2.7 (1.3-3.7)	0.70	98.2 (77.5-122.8)	0.36	
Non-JEE	78	7.1 (1.3-16.9)		11190.0 (5950.0-22890.0)		4.5 (3.6-6.3)		76.0 (73.6-81.1)		9.6 (4.1-16.5)		2.7 (1.3-5.5)		88.8 (42.7-120.0)		
**SEARO:**																
JEE	5	52.4 (21.0-68.7)	0.86	3840.0 (1210.0-5815.0)	0.17	2.0 (1.0-5.6)	0.92	74.6 (72.0-75.0)	0.10	12.3 (9.8-37.6)	0.27	5.8 (4.1-12.8)	0.20	24.7 (15.0-24.8)	0.92	
Non-JEE	6	27.0 (1.2-258.0)		1610.0 (1160.0-2610.0)		1.4 (1.3-2.3)		69.5 (68.5-70.0)		34.4 (27.2-47.7)		12.8 (9.6-15.8)		15.7 (13.4-27.5)		
**WPRO:**																
JEE	5	15.5 (6.7-51.0)	0.09	2160.0 (2100.0-3940.0)	0.11	2.6 (1.3-3.8)	0.04	69.8 (68.6-75.8)	0.62	22.4 (21.7-28.7)	0.61	6.4 (6.0-14.8)	0.28	24.1(11.2-65.6)	0.27	
Non-JEE	22	0.5 (0.1-15.9)		4920.0 (2970.0-30970.0)		4.6 (3.0-8.5)		73.4 (68.8-80.2)		20.0 (8.6-31.4)		3.3 (1.2-10.4)		46.8 (27.3-78.8)		

CD – communicable diseases, JEE – Joint External Evaluation, AFRO – Africa Regional Office, PAHO – Pan-American Health Organization, EMRO – Eastern Mediterranean Regional Office, SEARO – Southeast Asia Regional Office, EURO – European Regional Office, WPRO – Western Pacific Regional Office

*Millions of persons.

†United States dollars (US$).

‡Per 1000 live births.

§Reported in thousands of DALYs per 100 000 persons.

**Figure 1:**
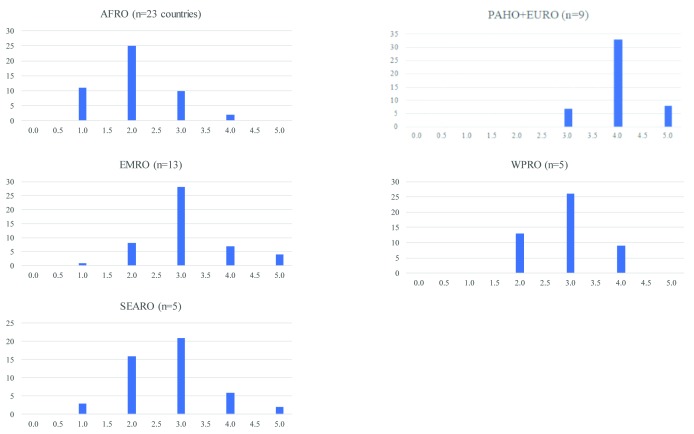
Distribution of median performance on 48 JEE Indicators across the 6 WHO regions.

Forty-three of the 48 (89.6%) total indicators included in the JEE assessment had a median score of <4 across the 50 countries analyzed. Analyzed by region, 46 (95.8%) indicators had a median score <4 in AFRO, 7 (14.6%) in PAHO+EURO, 37 (77.1%) in EMRO, 40 (83.3%) in SEARO, and 39 (81.3%) in WPRO (**Figure 1**).

The following indicators had the lowest median scores across the sample: antimicrobial stewardship activities, surveillance of infections caused by antimicrobial resistance (AMR), and the presence of biosecurity systems. The highest median indicator values were the presence of national vaccine access and delivery protocols, laboratory testing capabilities for priority diseases, and syndromic surveillance systems.

**Figure 2:**
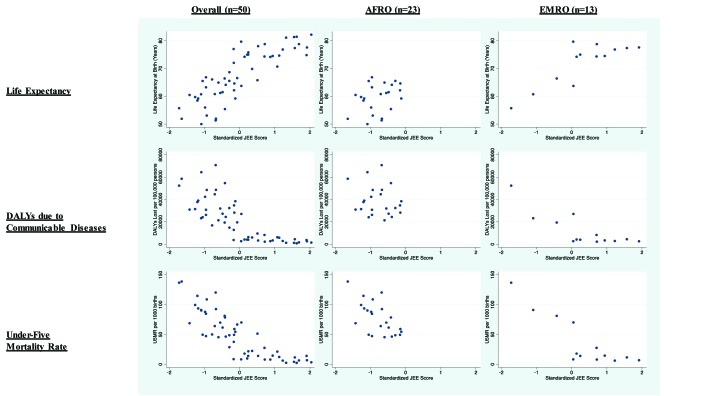
Scatter plots of various demographic characteristics with composite Joint External Evaluation (JEE) performance across various country groupings.

Statistically significant correlations were observed in the full sample between the JEE index score and life expectancy (Spearman’s ρ = 0.84, 95%CI = 0.76 to 0.90), DALYs lost to communicable diseases (ρ = -0.83, 95% CI = -0.90 to -0.75), and under-5 mortality rate per 1000 live births (ρ = -0.87, 95% = CI -0.92 to -0.78). In AFRO, the only statistically significant relationship with health outcomes was with the under-5 mortality rate (ρ = -0.59, 95% CI = -0.81 to -0.26) (**Table 3** and **Figure 2**). In EMRO, JEE performance was significantly correlated with under-five mortality (ρ = -0.80, 95% CI = 0.97 to -0.25) and lost DALYs to communicable diseases (ρ = -0.65, 95% CI = -0.92 to -0.01).

**Table 3:** Spearman rank-order correlation of various health outcomes and enabling factors with standardized score of JEE performance in the overall sample and stratified to the AFRO and EMRO regions

	Spearman’s Rho (95% confidence interval)*		
**Health or economic outcome**	**Overall (n = 55 countries)**	**AFRO (n = 23)**	**EMRO (n = 13)**
Life expectancy at birth (years)	0.84 (0.76 to 0.90)	0.34 (-0.05 to 0.66)	0.66 (-0.15 to 0.97)
Lost DALYs attributable to communicable diseases	-0.83 (-0.90 to -0.75)	-0.17 (-0.52 to 0.21)	-0.65 (-0.92 to -0.01)
Under-5 mortality rate (per 1000 live births)	-0.87 (-0.92 to -0.78)	-0.59 (-0.81 to -0.26)	-0.80 (-0.97 to -0.25)
**Enabling factors:**			
Gross domestic product per capita (US$)	0.81 (0.68 to 0.89)	0.17 (-0.25 to 0.55)	0.85 (0.61 to 0.99)
Public health (% of GDP)	0.36 (0.10 to 0.58)	0.10 (-0.33 to 0.49)	0.26 (-0.35 to 0.73)
Density of skilled health professionals	0.81 (0.71 to 0.89)	0.07 (-0.46 to 0.54)	0.51 (-0.15 to 0.90)

DALY – Disability-Adjusted Life Years, AFRO – Africa Regional Office, EMRO – Eastern Mediterranean Regional Office, GDP – Gross Domestic Product

*Bias-corrected and accelerated percentile 95% confidence intervals based on 10 000 bootstrap replications.

**Figure 3:**
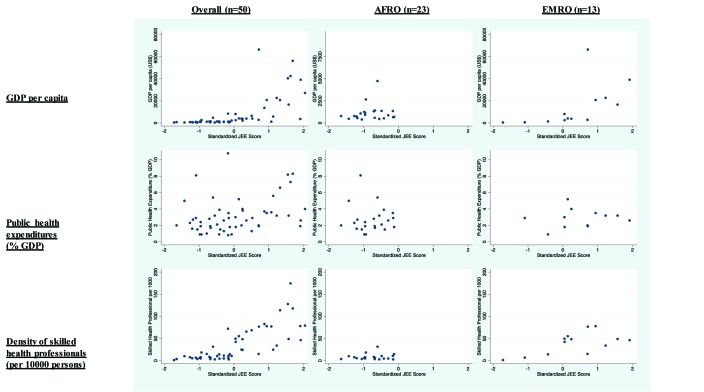
Scatter plots of various enabling factors with composite Joint External Evaluation (JEE) performance across various country groupings.

Among enabling factors, statistically significant correlations were observed within the overall sample for GDP per capita (ρ = 0.81, 95% CI = 0.68 to 0.89), public health expenditures as a percentage of GDP (ρ = 0.36, 95% = CI 0.10 to 0.58), and density of skilled health professionals per 10000 persons (ρ = 0.81, 95% = CI 0.71 to 0.89) (**Table 3** and **Figure 3**). No enabling factor was significantly correlated with JEE score in AFRO, but GDP per capita was correlated with JEE performance in EMRO (ρ = 0.85, 95% CI = 0.61 to 0.99).

## DISCUSSION

We examined performance on the Joint External Evaluation, the WHO’s voluntary tool for assessing IHR core capacity and preparedness to potential public health threats, among the initial 55 countries that have undergone the assessment. These evaluations reveal large gaps in core public health capacity and preparedness across a range of indicators with a large majority of countries scoring less than 4 (indicating non-sustainable or undeveloped capacities) on a majority of indicators. Antimicrobial stewardship and surveillance for highly resistant pathogens were areas of particular concern though there were other areas with substantial challenges as well. There were large variations in performance by geography, with countries from WHO AFRO and SEARO regions scoring lowest on the JEE and those in PAHO and EURO scoring highest. Taken together, these findings are a critical wake-up call that while JEE assessments are critical, they are only a first step towards making the kinds of financial and technical investments necessary to help countries improve their public health capacities.

Most of the capacities measured by the indicators in the JEE represent essential public health functions, such as disease surveillance, laboratory capacity, and sufficient human resources. These combine to create an infrastructure which protects the health of a country’s population from threats such as communicable zoonotic diseases, food poisoning and waterborne illnesses, allowing nations to contain public health emergencies at their source. The poor performance on many of these indicators (scores of less than 4) suggest that countries are not only ill-prepared for cross-border outbreaks but are struggling to provide key public health services that are critical to keeping their populations healthy and safe. Without these core capacities in place, future outbreaks may become large-scale pandemics. Reasons for poor capacity are clearly multifactorial, as many countries exhibit broad-based weaknesses across a range of indicators. One fact is clear from these analyses: further investments are needed that specifically target preparedness and core public health functions represented by the JEE.

Yet, success in building any capacity can only be achieved with leadership and investment of resources, prioritization, and commitment by the specific countries themselves. Few countries completely lack capacity but appear to have some existing infrastructure and competence on which to build. Not surprisingly, resource-constrained regions of the world performed less well on the JEE than relatively affluent regions like PAHO, EURO and parts of EMRO and WPRO. Further, poor health outcomes, as measured by DALYs due to communicable disease, are more frequent in AFRO and SEARO, which have high burdens of disease while also exhibiting lower life expectancy and higher mortality rates. The observation that JEE performance seems to correlate well with these baseline health, demographic and economic data points suggests that this tool is accurately measuring its intended core public health-related capabilities.

Indeed, the JEE does appear to correlate well with the gross overview of the strength and performance of the core public health components of health systems as defined by life expectancy, under 5-year old mortality, and disability due to communicable disease. Moreover, performance on the JEE positively correlated with the presence of enabling resources for health (high levels of GDP per capita and skilled health professionals). However, less consistent correlations in region-specific analyses highlight a need to further validate the JEE tool across contexts and suggests value for context-specific prioritization of functions. Overall, our findings suggest the JEE tool accurately measures relevant health security capabilities, though longitudinal studies will be needed to confirm this. A slightly revised update of the tool was recently published by the WHO, but changes are small and thus unlikely to affect our results. So far, no JEE reports using the revised tool have been published.

There are important limitations to our work. First, JEE reports have only been conducted on 28.2% (n = 55) of the WHO Member States; further, each evaluated country has had only one assessment. Also, for some specific activities, such as prevention of antimicrobial drug resistance, interventions have started only relatively recently. Thus, the limitations of a cross-sectional analysis apply here and diminish the generalizability of our findings, since we expect variation in JEE performance over time to be more informational in guiding improvement. Further, there is asymmetric representation from each WHO region, which may have led to an inaccurate estimation of regional trends in JEE performance; however, with our adjustments, we believe this initial analysis will provide helpful guidance to policymakers as an interim summary on JEE results to-date.

Moreover, our analysis is not intended to serve as a definitive validation exercise, nor are we aiming to assess cause and effect between JEE scores and outcomes. Our relatively small data set handicapped our ability to pursue more advanced statistical models that may better identify possible predictors of JEE performance. As more analyses are performed, and particularly as countries go through additional iterations of the assessment over time, we believe pursuing such analyses in the future will be both helpful and appropriate.

In conclusion, core public health functions and preparedness remains broadly weak across a range of indicators among a moderately large sample of WHO member countries, though substantial regional variations exist. Capabilities in AMR and emergency response are among the lowest scoring, and our results suggest that regions with greater health resources exhibit improved performance on the JEE. We do find, however, that the JEE tool itself and the process it is applied by are important and seemingly effective in measuring the strength of the core public health components of health systems. As global generic indicators for these functions remain sparse and underutilized, the JEE process provides an additional opportunity to measure the impact of investing in essential public health functions.
