# Supplementary material for: Analysis of results from the Joint External Evaluation: examining its strength and assessing for trends among participating countries
Source: J Glob Health. 2018 Aug 23;8(2):020416. doi: 10.7189/jogh.08.020416 (PMC6204750; doi:10.7189/jogh.08.020416)
Supplement: Online Supplementary Document [file jogh-08-020416-s001.pdf]

Online Supplementary Document

Gupta et al. Analysis of results from the Joint External Evaluation: examining its strength and assessing for trends among participating countries

J Glob Health 2018;8:020416

Table S1. Listing of all countries to complete and publish the results of their JEE as of March 4, 2018.

Afghanistan	Mauritania
Albania	Mongolia
Armenia	Morocco
Bahrain	Mozambique
Bangladesh	Myanmar
Belgium	Namibia
Benin	Nigeria
Cambodia	Oman
Cameroon	Pakistan
Chad	Qatar
Côte d'Ivoire	Saudi-Arabia
Eritrea	Senegal
Ethiopia	Sierra Leone
Finland	Slovenia
Gambia	Somalia
Ghana	Sri Lanka
Guinea	Sudan
Jordan	Tanzania (United Republic of)
Kenya	Thailand
Korea (Republic of)	Tunisia
Kyrgystan	Turkmenistan
Lao People's Democratic Republic	Uganda
Latvia	United Arab Emirates
Lebanon	United States of America
Lesotho	Vietnam
Liberia	Zambia
Madagascar	
Maldives	
Mali	

Table S2. Indicators across the full JEE assessment.

<u>Full JEE Assessment:</u> 48 total indicators

Capacities	Indicators
**National legislation, policy and financing**	P.1.1 Legislation, laws, regulations, administrative requirements, policies or other government instruments in place are sufficient for implementation of IHR (2005)
	P.1.2 The State can demonstrate that it has adjusted and aligned its domestic legislation, policies and administrative arrangements to enable compliance with IHR (2005)
**IHR coordination, communication and advocacy**	P.2.1 A functional mechanism is established for the coordination and integration of relevant sectors in the implementation of IHR
**Antimicrobial resistance**	P.3.1 Antimicrobial resistance detection
	P.3.2 Surveillance of infections caused by antimicrobial-resistant pathogens
	P.3.3. Health care-associated infection (HCAI) prevention and control programs
	P.3.4 Antimicrobial stewardship activities
**Zoonotic Diseases**	P.4.1 Surveillance systems in place for priority zoonotic diseases/pathogens
	P.4.2 Veterinary or animal health workforce
	P.4.3 Mechanisms for responding to infectious and potential zoonotic diseases are established and functional
**Food Safety**	P.5.1 Mechanisms are established and functioning for detecting and responding to foodborne disease and food contamination.
**Biosafety and biosecurity**	P.6.1 Whole-of-government biosafety and biosecurity system is in place for human, animal and agriculture facilities
	P.6.2 Biosafety and biosecurity training and practices
**Immunization**	P.7.1 Vaccine coverage (measles) as part of national programme
	P.7.2 National vaccine access and delivery
**National laboratory system**	D.1.1 Laboratory testing for detection of priority diseases
	D.1.2 Specimen referral and transport system

	D.1.3 Effective modern Point-of-care and laboratory-based diagnostics
	D.1.4 Laboratory quality system
**Real-time surveillance**	D.2.1 Indicator and event-based surveillance systems
	D.2.2 Interoperable, interconnected, electronic real-time reporting systems
	D.2.3 Analysis of surveillance data
	D.2.4 Syndromic surveillance systems
**Reporting**	D.3.1 System for efficient reporting to WHO, FAO and OIE
	D.3.2 Reporting network and protocols in country
**Workforce Development**	D.4.1 Human resources available to implement IHR core capacity requirements
	D.4.2 Applied epidemiology training program<br>in place such as FETP
	D.4.3 Workforce strategy
**Preparedness**	R.1.1 Multi-hazard national public health emergency preparedness and response plan is developed and implemented
	R.1.2 Priority public health risks and resources are mapped and utilized
**Emergency response operations**	R.2.1 Capacity to activate emergency operations
	R.2.2 EOC operating procedures and plans
	R.2.3 Emergency operations program
	R.2.4 Case management procedures implemented for IHR relevant hazards
**Linking public health and security authorities**	R.3.1 Public health and security authorities (e.g. law enforcement, border control, customs) are linked during a suspect or confirmed biological event
**Medical Countermeasures and personnel deployment**	R.4.1 System in place for sending and receiving medical countermeasures during a public health emergency
	R.4.2 System in place for sending and receiving health personnel during a public health emergency
**Risk Communication**	R.5.1 Risk communication systems (plans, mechanisms, etc.)
	R.5.2 Internal and partner communication and coordination
	R.5.3 Public communication
	R.5.4 Communication engagement with affected communities

	R.5.5 Dynamic listening and rumor management
**Points of entry (PoE)**	PoE.1 Routine capacities established at points of entry
	PoE.2 Effective public health response at points of entry
**Chemical Events**	CE.1 Mechanisms established and functioning for detecting and responding to chemical events or emergencies
	CE.2 Enabling environment in place for management of chemical events
**Radiation emergencies**	RE.1 Mechanisms are established and functioning for detecting and responding to radiological and nuclear emergencies
	RE.2 Enabling environment in place for management of radiation emergencies

Appendix S3. Definitions of each category score across all JEE indicators.

Every indicator in the JEE evaluation tool is scored based on a county's capacity at the time of evaluation. Scores range from 1-5 for all indicators and a country receives a single score for each indicator.

Evaluators are equipped with both Contextual and Technical Area Questions to help determine the appropriate score. For consistency and ease of use, definitions of each score option (1 – 5) are the same across all indicators (see "JEE Tool, General"). However, the JEE tool does outline example text to help evaluators understand how scoring applies for each specific indicator (e.g. AMR detection, below). To provide context, information on overall "Target" and "Desired Impact" are also provided.

Score	JEE Tool, General	AMR Example
1. No Capacity	Attributes of a capacity are not in place.	No national plan for detection and reporting of priority AMR pathogens has been approved
2. Limited Capacity	Attributes of a capacity are in development stage (some are achieved and some are undergoing; however, the implementation has started).	National plan for detection and reporting of priority AMR pathogens has been approved
3. Developed Capacity	Attributes are in place, sustainable for a few more years and can be measured by the inclusion of attributes or IHR (2005) core capacities in the national health sector plan	Designated laboratories are conducting detection and reporting of some priority AMR pathogens
4. Demonstrated Capacity*	Attributes are in place, sustainable for a few more years and can be measured by the inclusion of attributes or IHR (2005) core capacities in the national health sector plan.	Designated laboratories have conducted detection and reporting of all priority AMR pathogens for at least one year
5. Sustainable Capacity	Attributes are functional, sustainable and the country is supporting other countries in its implementation. This is the highest level of the achievement of implementation of IHR (2005) core capacities.	Designated laboratories have conducted detection and reporting of all priority AMR pathogens for years with a system for continuous improvement

*in order to reach demonstrated capacity, one has to meet all the attributes of previous scores.

ANTIMICROBIAL RESISTANCE (Example Target & Desired Impact)

Target: Support work being coordinated by WHO, FAO, and OIE to develop an integrated global package of activities to combat antimicrobial resistance, spanning human, animal, agricultural, food and environmental aspects (i.e. a one-health approach), including: a) Each country has its own national comprehensive plan to combat antimicrobial resistance; b) Strengthen surveillance and laboratory capacity at the national and international level following agreed international standards developed in the framework of the Global Action plan, considering existing standards and; c) Improved conservation of existing treatments and collaboration to support the sustainable development of new antibiotics, alternative treatments, preventive measures and rapid, point-of-care diagnostics, including systems to preserve new antibiotics. As Measured by: (1) Number of comprehensive plans to combat antimicrobial resistance agreed and implemented at a national level, and yearly reporting against progress towards implementation at the international level. (2) Number of countries actively participating in a twinning framework, with countries agreeing to assist other countries in developing and implementing comprehensive activities to combat antimicrobial resistance, including use of support provided by international bodies to improve the monitoring of antimicrobial usage and resistance in humans and animals.

Desired Impact: Decisive and comprehensive action to enhance infection prevention and control activities to prevent the emergence and spread of AMR, especially among drug-resistant bacteria. Nations will strengthen surveillance and laboratory capacity; ensure uninterrupted access to essential antibiotics of assured quality; regulate and promote the rational use of antibiotics in human medicine and in animal husbandry and other fields as appropriate; and support existing initiatives to foster innovations in science and technology for the development of new antimicrobial agents.
